# Developmental MRI markers cosegregate juvenile patients with myoclonic epilepsy and their healthy siblings

**DOI:** 10.1212/WNL.0000000000008173

**Published:** 2019-09-24

**Authors:** Britta Wandschneider, Seok-Jun Hong, Boris C. Bernhardt, Fatemeh Fadaie, Christian Vollmar, Matthias J. Koepp, Neda Bernasconi, Andrea Bernasconi

**Affiliations:** From the Neuroimaging of Epilepsy Laboratory (B.W., S.-J.H., B.C.B., F.F., N.B., A.B.), McConnell Brain Imaging Center, Montreal Neurological Institute, McGill University, Montreal; Department of Clinical and Experimental Epilepsy (B.W., C.V., M.J.K.), UCL Institute of Neurology, London, UK; Epilepsy Center, Department of Neurology (C.V.), Klinikum Großhadern, University of Munich, Germany; and Multimodal Imaging and Connectome Analysis Lab (B.C.B.), Montreal Neurological Institute and Hospital, McGill University, Montreal, Canada.

## Abstract

**Objective:**

MRI studies of genetic generalized epilepsies have mainly described group-level changes between patients and healthy controls. To determine the endophenotypic potential of structural MRI in juvenile myoclonic epilepsy (JME), we examined MRI-based cortical morphologic markers in patients and their healthy siblings.

**Methods:**

In this prospective, cross-sectional study, we obtained 3T MRI in patients with JME, siblings, and controls. We mapped sulco-gyral complexity and surface area, morphologic markers of brain development, and cortical thickness. Furthermore, we calculated mean geodesic distance, a surrogate marker of cortico-cortical connectivity.

**Results:**

Compared to controls, patients and siblings showed increased folding complexity and surface area in prefrontal and cingulate cortices. In these regions, they also displayed abnormally increased geodesic distance, suggesting network isolation and decreased efficiency, with strongest effects for limbic, fronto-parietal, and dorsal-attention networks. In areas of findings overlap, we observed strong patient–sibling correlations. Conversely, neocortical thinning was present in patients only and related to disease duration. Patients showed subtle impairment in mental flexibility, a frontal lobe function test, as well as deficits in naming and design learning. Siblings' performance fell between patients and controls.

**Conclusion:**

MRI markers of brain development and connectivity are likely heritable and may thus serve as endophenotypes. The topography of morphologic anomalies and their abnormal structural network integration likely explains cognitive impairments in patients with JME and their siblings. By contrast, cortical atrophy likely represents a marker of disease.

Generalized genetic epilepsies, previously known as primary generalized or idiopathic generalized epilepsies, refer to a group of syndromes with known or presumed genetic origin, with juvenile myoclonic epilepsy (JME) being most common. The syndrome's clinical hallmark is myoclonus. Most patients also experience generalized tonic-clonic seizures and more rarely absences.^1^ JME is associated with cognitive impairment, mainly affecting frontal lobe function, together with emotional instability and psychiatric comorbidities.^2^ While visual MRI analysis does not reveal obvious structural abnormalities,^2^ morphometric studies comparing patients to healthy individuals have shown evidence for subtle cortico-subcortical structural derangements, particularly in the thalamus and frontal lobe.^2–4^ Notably, first-degree relatives of patients with JME are at risk for epilepsy and may present with similar cognitive traits, supporting genetic underpinning.^5,6^ Notwithstanding a complex polygenetic inheritance suspected in most patients, mutations in the *EFHC1* gene have shown to be disease-causative.^7^ In experimental models, loss of function of this gene regulating cell division and migration leads to disrupted corticogenesis,^8^ which may explain abnormal cortical lamination observed in human postmortem studies.^9^

MRI lends metrics to study the interplay among brain structure, genes, and environment, thereby providing opportunities to assess endophenotypes; that is, the intermediate traits more closely related to the genetic makeup than clinical manifestations.^10^ An important characteristic of an endophenotype is its presence in unaffected family members at a higher rate than in the general population; notably, studying asymptomatic sibling controls for disease activity and medication effects while ensuring comparable age and environmental factors.^11^ In psychiatric disorders, neuroimaging-derived endophenotypes have successfully mapped effects of a number of genetic variants, necessitating smaller samples than those required in traditional case–control studies.^12^ In epilepsy, most effort has been dedicated to exploring MRI endophenotypes in focal syndromes, primarily temporal lobe epilepsy.^13,14^ Conversely, structural MRI studies of JME have so far described group-level changes between patients and healthy controls, which have been interpreted as disease effect.^2^

Our purpose was to assess the endophenotypic potential of structural MRI in JME. We studied cohorts of patients with JME, unaffected siblings, and healthy controls and computed cortical thickness, sulco-gyral complexity, and surface area, quantitative imaging markers tapping into complementary aspects of cortical morphology and thought to have different genetic underpinnings.^15^ Morphometric assessments were complemented by geodesic distance mapping, a recently proposed surrogate marker of “wiring cost” that may serve as a measure of intrinsic cortico-cortical connectivity.^16^

## Methods

### Participants

In this cross-sectional study, we studied 29 consecutive patients with JME, 16 unaffected siblings (related to 11 patients), and 20 healthy controls. Patients were recruited from epilepsy outpatient clinics at University College London Hospitals between 2007 and 2013. Siblings were contacted with the consent of the related patient. Groups were comparable for age (mean ± SD: patients = 35.7 ± 11.1; siblings = 38.9 ± 13.0; controls = 32.6 ± 8.5; analysis of variance [ANOVA], *p* = 0.2) and sex (male: 41%, 38% siblings, 30% controls; χ^2^ = 0.66, *p* = 0.7). All participants underwent a structured interview in regard to chronic neurologic conditions and a brief neurologic examination. Healthy controls and unaffected siblings had no significant neurologic history and all participants had a normal neurologic examination. In all participants, MRI scans were reviewed by a neuroradiologist and reported as normal. Patients had a typical history of JME with myoclonic jerks, generalized tonic-clonic seizures, and absence seizures in one third (10/29), with disease onset at 14.0 ± 3.6 years. At the time of the MRI acquisition, disease duration was 22.1 ± 11.8 years and time since last seizure was 1,031.5 ± 1,379.9 days; patients were treated on average with 1.6 ± 0.64 antiepileptic drugs. In all, routine scalp EEG showed generalized polyspike wave complexes. No sibling had ever experienced unprovoked seizures.

All participants underwent neuropsychological testing on the day of scanning, which evaluated verbal IQ, verbal comprehension and expressive language, verbal and nonverbal learning, and psychomotor speed. For higher frontal lobe functions, we assessed working memory, mental flexibility, and fluency (table).

**Table:**
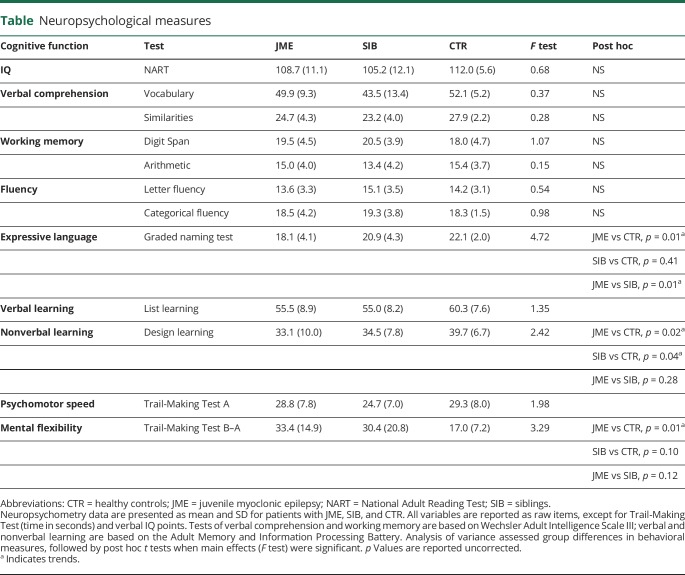
Neuropsychological measures

### Standard protocol approvals, registrations, and patient consents

The Ethics Committee of the University College London Institute of Neurology and University College London Hospitals approved the study and written informed consent was obtained from all participants in accordance with the standards of the Declaration of Helsinki.

### MRI acquisition and image processing

T1-weighted structural MRI data were obtained on a 3T General Electric (Boston, MA) Excite HD scanner using a 3D fast spoiled gradient echo (repetition time 7.2 ms, echo time 2.8 ms, inversion time 450 ms, flip angle = 20°, matrix = 256×256, 176 sagittal slices, voxel size = 1.1×1.1×1.1 mm^3^). MRI data were processed using FreeSurfer (surfer.nmr.mgh.harvard.edu/; version 5.3). Preprocessing included bias field correction, registration to stereotaxic space, intensity normalization, skull-stripping, and hemispheric white matter segmentation. A triangular surface tessellation fitted a deformable mesh model onto the white matter volume, providing gray–white and pial surfaces with >160,000 corresponding points (or vertices). Individual surfaces were registered to an average template surface with a spherical representation, improving the correspondence of measurement points with regards to sulcation. Surface extractions were visually verified, and topologic defects manually corrected.

### Computation of morphologic features

#### Cortical thickness

The thickness of the cortex reflects various cellular-level features including size, density, and arrangement of neurons, as well as neuroglia and nerve fibers.^17^ We measured thickness as the distance of corresponding vertices between the gray–white matter and pial boundaries.

#### Curvature

Sulco-gyral folding of the neocortex occurs primarily between the 26th and 36th week of gestation.^18^ To quantify cortical folding complexity, we estimated mean curvature by computing the maximum and minimum principal curvatures (i.e., inverse of radius of an inscribed circle), and averaging them at each vertex between the pial and gray–white matter interface. This analysis was carried out using mris_curvature, a built-in Freesurfer function. As curvature might be affected by variations of cortical thickness, we statistically adjusted this metric at every vertex by the corresponding thickness measure.^19^

#### Surface area

Surface area is thought to reflect the relative expansion or compression of cortical columns within a given area.^20^ As in previous work,^16^ we measured the average surface area determined by 6 triangular meshes surrounding each vertex along the gray–white matter interface. To account for interpolation effects during surface registration, we computed this metric based on the surface resampled to the template (i.e., fsaverage).^21^ Measurements were corrected for total white matter volume.

### Evaluation of cortico-cortical connectivity

We measured geodesic distance, representing the shortest path between any 2 points (or surface vertices) along the cortical mantle, a feature that has been related to intrinsic cortico-cortical connectivity.^16^ As in previous work, this metric was calculated using an approach invariant to mesh configuration.^22^ For each vertex, we calculated the average distance to all other vertices, generating a mean geodesic distance map per individual. The resulting distances were corrected for total white matter volume.^16^

### Statistical analysis

Statistical analysis was carried out using SurfStat for MATLAB (math.mcgill.ca/keith/surfstat/)^23^. Prior to analysis, surface-based measurements were blurred using a diffusion kernel (full width at half maximum = 20 mm) that respects surface topology and *z*-normalized at each surface point with respect to the corresponding distribution in healthy controls.

Surface-wise Student *t* tests separately compared curvature, surface area, cortical thickness, and geodesic distance between patients with JME and controls, and between siblings and controls. Given that curvature and surface area are markers of brain development, to signify the overall load of anomalies, we repeated the above comparisons based on their multivariate combination using the Hotelling *t* test. To address possible confounds of gray matter atrophy, we repeated the analysis of curvature and surface area, after statistically controlling for thickness at each surface point. Analyses were corrected using random field theory,^24^ controlling the family-wise error at *p*_FWE_ < 0.05.

### Data availability

Surface-based features for all analyses are available upon request.

## Results

### Analysis of morphologic markers

**Figure 1:**
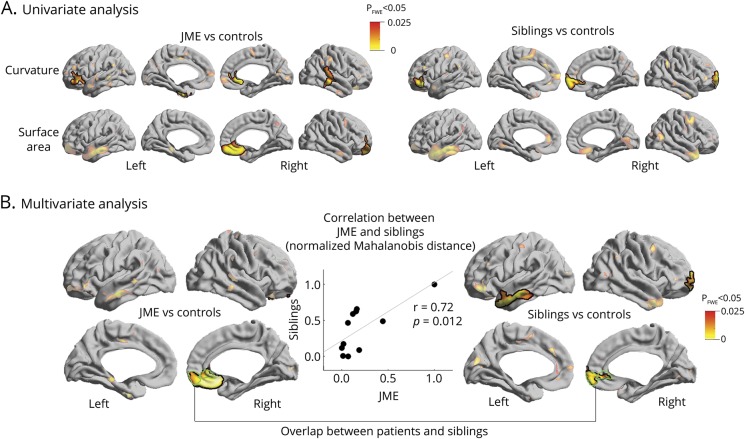
Analysis of curvature and surface area (A) Univariate group analysis shows regions of increased curvature and surface area comparing patients with juvenile myoclonic epilepsy (JME) to controls, and siblings to controls. (B) Multivariate analysis assesses the joint distribution of curvature and surface area. In regions of overlap (outlined in green), linear models show positive correlation between each patient and his or her siblings. Significant clusters corrected for multiple comparisons using random field theory at *p*_FWE_ < 0.05 are outlined in black.

**Figure 2:**
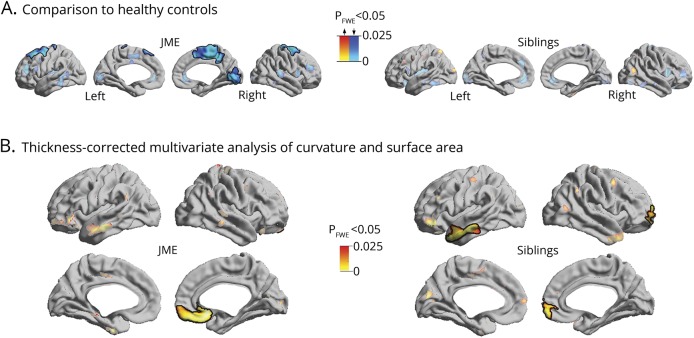
Analysis of cortical thickness (A) Group analysis shows regions of neocortical thinning in patients with juvenile myoclonic epilepsy (JME) compared to controls, while no changes are observed between siblings and controls. (B) Thickness-corrected multivariate analysis of curvature and surface area show same pattern of anomalies as those observed in figure 1B. Significant clusters corrected for multiple comparisons using random field theory at *p*_FWE_ < 0.05 are outlined in black.

Compared to healthy participants, patients with JME and siblings exhibited increased curvature and surface area in orbitofrontal, anterior cingulate, and temporal cortices (*p*_FWE_ < 0.05; figure 1A). In areas of overlap, multivariate linear models directly assessed the relationship between each patient and his or her siblings, showing marked patient–sibling correlation (*r* = 0.72, *p* = 0.012; figure 1B). The analysis of cortical thickness revealed thinning of fronto-central and occipital cortices in patients with JME compared to healthy participants (*p*_FWE_ < 0.03; figure 2A), while no differences were observed between siblings and controls. Repeating the univariate and multivariate analysis of curvature and surface area, while controlling for cortical thickness variations at each surface point, did not modify results (figure 2B).

### Analysis of geodesic distance

**Figure 3:**
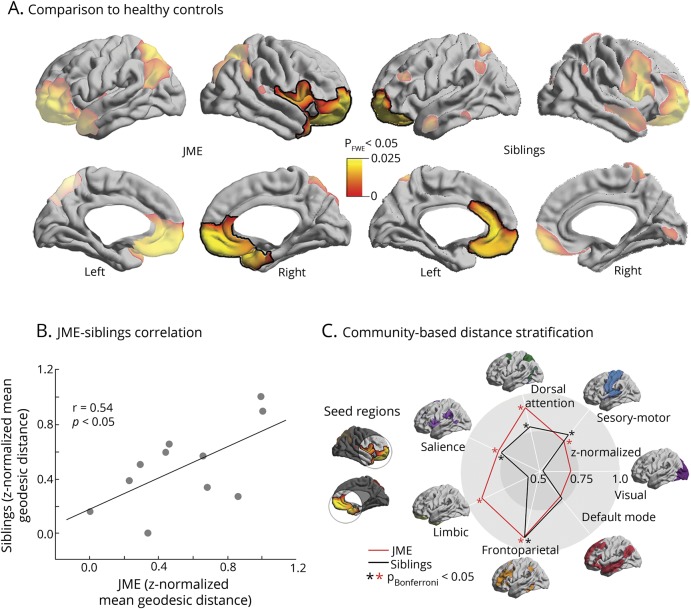
Analysis of geodesic distance (A) Univariate group analysis shows regions of increased mean geodesic distance between patients with juvenile myoclonic epilepsy (JME) and controls, and siblings and controls. Significant clusters corrected for multiple comparisons using random field theory at *p*_FWE_ < 0.05 are outlined in black. (B) In regions of overlap, linear models show positive correlation of geodesic distance (*z*-normalized with respect to the corresponding distribution in healthy controls) between each patient and his or her siblings. (C) Spider plots display the *z*-normalized distance from clusters of significant differences to functional networks relative to healthy controls. Cohen *d* effect sizes of group differences for each target network in patients and siblings are as follows: visual (0.6/0.4), sensorimotor (0.8/0.7), dorsal attention (0.9/0.7), salience (0.8/0.7), limbic (0.8/0.5), fronto-parietal (0.8/0.7), default mode (0.6/0.5).

Relative to controls, both patients with JME and siblings showed increases in mean geodesic distance in prefrontal (orbitofrontal, ventrolateral, inferior premotor), anterior cingulate, as well as temporo-polar cortices to the remaining neocortex, suggesting decreased network efficiency (*p*_FWE_ < 0.05; figure 3A). In areas of overlap, linear models revealed a significant patient–sibling relationship (*r* = 0.54, *p* < 0.05; figure 3B).

In a post hoc analysis (figure 3C), we computed the geodesic distance between the clusters of findings and each of 7 well-established functional networks (i.e., visual, sensorimotor, dorsal attention, salience, limbic, frontoparietal, and default mode networks).^25^ For each target network, a linear model compared groups. In JME, we found increased geodesic distance from the clusters of findings to all canonical networks, except to the visual and default mode network (*p* < 0.05, Bonferroni-corrected); most marked effects were seen in limbic, fronto-parietal, and dorsal attention networks. Except for limbic and visual networks, siblings showed similar effects (*p* < 0.05, Bonferroni-corrected).

### Assessing specificity of MRI endophenotypes

To test the robustness of the observed endophenotypes (i.e., curvature, surface area, and geodesic distance), and dispel possible low-level intersubject correlation, we used permutation testing. To this end, we randomly selected 2 groups of healthy controls, each with the same sample size as patients with JME and their siblings (n = 11) and repeated the correlation analyses. Across 1,000 iterations, neither the multivariate combination of curvature and surface area nor geodesic distance showed correlation coefficients higher than in the original analyses, further supporting their significance as endophenotypes.

### Clinical correlation analysis

With respect to neuropsychological testing, ANOVA showed main effects of groups on expressive language (*F* = 4.7, *p* = 0.01), mental flexibility (*F* = 3.3, *p* = 0.04), and trends for design learning (*F* = 2.4, *p* = 0.09). Post hoc comparisons revealed slightly poorer performance of these tests for JME compared to controls than siblings compared to controls, but results remained at a trend level after correcting for multiple comparisons (table).

**Figure 4:**
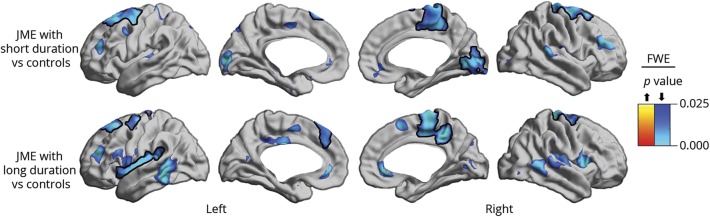
Duration-stratified cortical thickness group analysis Patients were split into short and long duration subgroups according to the median (16 years) and compared to controls. Cortical thickness decreases are shown in blue. Significant clusters, corrected for multiple comparisons using random field theory at family-wise error (FWE) < 0.05, are shown in solid colors and outlined in black; trends are shown in semitransparence. JME = juvenile myoclonic epilepsy.

To explore patients' heterogeneity, we subdivided them with respect to median disease duration (16 years) into short (n = 16) and long (n = 13). We found that patients with a median disease duration >16 years had more widespread cortical thinning, particularly in limbic cortices, including the insula and cingulate (figure 4). However, there was no significant difference for lifetime medication load (i.e., total number of antiepileptic agents over disease course; mean and SD: 3.5 ± 2.2 vs 3.6 ± 1.7; *t* = −0.17, *p* = 0.9) and days since last seizure (1,134 ± 1,493 vs 653 ± 881; *t* = 1.01, *p* = 0.3) when comparing short to long duration, respectively.

## Discussion

We assessed the endophenotypic potential of structural MRI in JME by analyzing surface-based morphology and cortico-cortical connectivity in patients and their unaffected siblings. Both cohorts showed increased curvature and surface area in prefrontal and cingulate cortices. In these regions, they also displayed abnormally increased geodesic distance, suggesting network isolation and decreased efficiency, with strongest effects for limbic, fronto-parietal, and dorsal-attention networks. Altogether, our results indicate that these MRI markers of brain development^15,18,19^ and connectivity are likely heritable and thus relate to the underlying disease neurobiology. Strong patient–sibling correlations, in addition verified with permutation testing, further support their role as endophenotypes. By contrast, cortical atrophy likely represents a marker of disease.

Increased sulco-gyral complexity has been documented in mild forms of cortical dysplasia and polymicrogyria, developmental malformations thought to arise during late stages of corticogenesis.^26^ Surface area, a marker of cortical expansion, has been linked to early tangential migration of neurons,^27^ as well as postnatal local synaptogenesis, cortical myelination, and dendritic arborization.^28^ Thus, our findings of increased curvature and surface area likely result from abnormal post-migrational development. Notably, these anomalies were mainly localized in prefrontal and cingulate cortices, higher-order polymodal association regions known to undergo late maturation.^29^ Late developmental stages are critical for system-level network organization and area specialization through formation of intrahemispheric and interhemispheric fiber connections.^30^ Disruptions at this stage likely affect regional integration and segregation, which may represent a mechanistic explanation for aberrant structural and functional connectivity between primary motor and cognitive networks observed in JME.^4,31^

Geodesic distance has been proposed as a measure of global wiring costs pertaining to intrinsic (i.e., cortico-cortical) synaptic connections and serves as a proxy for large-scale network organization.^16^ Short connectional length minimizing wiring costs is associated with increased network efficiency.^32^ In vitro data and in vivo MRI studies have implicated abnormal intrinsic connections in several neurodevelopmental conditions.^16,33^ In our patients with JME, increased mean geodesic distance in the prefrontal, cingulate, and temporopolar cortices may ultimately translate into atypical cortico-cortical connectivity, with additional wiring costs needed to maintain function. While anomalies were more severe in patients, siblings showed a similar degree and topography of changes relative to controls. As demonstrated in the post hoc analysis, these regions appear to be relatively isolated from the rest of the brain and exhibit increased distance to virtually all functional networks, with more prominent effects observed for limbic, fronto-parietal, and dorsal attention networks. This may relate to less efficient information transfer contributing to abnormal cortico-subcortical connectivity observed in JME.^4^ Moreover, our findings dovetail with functional imaging, demonstrating abnormal connectivity between motor and fronto-parietal cognitive networks in patients with JME and their siblings.^31,34^

The predominant topography of morphologic anomalies in prefrontal cortices and their abnormal structural network integration with limbic, fronto-parietal, and dorsal attention likely explains the cognitive impairments reported in patients with JME^34–36^ and, to a lesser extent, their siblings,^5,34^ and are possibly related to altered developmental trajectories.^37^ Indeed, these regions are critical hubs for higher-order processes, including working and prospective memory,^38^ emotional processing,^39^ impulsivity, and risk-taking behavior.^40^ In JME, sociocognitive dysfunction is known to contribute to “real-life problems” including treatment incompliance, social impulsiveness, and poor psychosocial outcomes, such as unemployment and social isolation.^2^ These deficits are often present despite good seizure control.^41^

In line with previous literature,^42^ our JME cohort showed subtle impairment in mental flexibility, a frontal lobe function test. Deficits beyond frontal lobe functions, such as naming and design learning observed here, have also been reported^42^ and underline heterogeneity. For mental flexibility, siblings' performance fell between patients and controls. Similar results have been shown in previous studies on JME siblings,^5,6,42^ supporting the notion that these cognitive traits are heritable and part of the broader disease phenotype. Our study did not have institution approval to include EEG recordings in asymptomatic siblings. Subclinical EEG anomalies may be present in about 30% of unaffected siblings in the absence of a history of unprovoked seizures,^43^ suggesting that epileptiform discharges, similar to cognitive traits, may be part of a wider disease spectrum.

While anomalies of curvature and surface are suggestive of late, abnormal postmigrational processes, atypical geodesic distance may reflect early disruption. This is supported by experimental models in *EFHC1/Myoclonin1* gene variants accounting for about 8% of JME cases.^7^ Mutations in *EFHC1*, a microtubule-associated protein, lead to disruption of cell proliferation and neuronal migration,^8^ possibly correlating with reports of mild malformations of cortical development in this condition.^9^ Atypical cortico-cortical network integration, as supported by increases in cortical geodesic distance, may thus mirror findings from gene-level experimental models demonstrating abnormal connections through overgrown dendrites with expanded synapse complexity.^44^ Moreover, excessive spontaneous transmitter release^45^ could be responsible for hyperexcitability, underscoring disease causality of *EFHC1* gene variants.^7^

In our study, atrophy in fronto-central and occipital cortices was only present in patients with JME but not in their siblings. Similarly, previous work in temporal lobe epilepsy failed to identify MRI-derived cortical thickness changes as a structural endophenotype, while other morphologic features, including localized contractions of cerebral surface area, showed correlated topologic alterations between patients and their first-degree relatives.^14,46^ In healthy people, cortical thickness is believed to be largely determined by prenatal and postnatal developmental processes.^47^ Progressive cortical atrophy associated with longer disease duration and increased seizure frequency, a phenomenon often reported in epilepsy, likely represents a marker of disease severity.^48^ Similarly, post hoc analysis in our study demonstrated more widespread cortical thinning, particularly involving limbic cortices, in patients with longer disease duration compared to controls, further supporting a modulatory effect of disease severity on cortical thickness.

In JME, previous work has shown variable patterns of increased^3^ or reduced cortical thickness^49^; disparities with our study may relate to differences in age, disease duration, and clinical phenotypes. Notably, fronto-central cortical anomalies have been related to cognitively triggered myoclonus.^31^ Further evidence that fronto-central atrophy may be disease-defining stems from studies in epileptic baboons, a natural JME model. Though baboons were sacrificed after short disease duration with sporadic seizures and no antiepileptic medication, neuronal loss was most pronounced in the primary motor cortex, particularly the hand area.^50^ This may represent reduced axonal and dendritic connections and U-fibers leading to local hyperexcitability, ultimately facilitating myoclonic jerks in a JME-typical somatotopic distribution.

## Glossary

ANOVA: analysis of variance
JME: juvenile myoclonic epilepsy

## References

[1] Kasteleijn-Nolst Trenité DGA, Schmitz B, Janz D, et al. Consensus on diagnosis and management of JME: from founder's observations to current trends. Epilepsy Behav 2013;28(suppl 1):S87–S90. 23756490 10.1016/j.yebeh.2012.11.051

[2] Koepp MJ, Thomas RH, Wandschneider B, Berkovic SF, Schmidt D. Concepts and controversies of juvenile myoclonic epilepsy: still an enigmatic epilepsy. Expert Rev Neurother 2014;14:819–831. 24931665 10.1586/14737175.2014.928203

[3] Alhusaini S, Ronan L, Scanlon C, et al. Regional increase of cerebral cortex thickness in juvenile myoclonic epilepsy. Epilepsia 2013;54:e138–e141. 23944956 10.1111/epi.12330

[4] O'Muircheartaigh J, Vollmar C, Barker GJ, et al. Abnormal thalamocortical structural and functional connectivity in juvenile myoclonic epilepsy. Brain 2012;135:3635–3644. 23250883 10.1093/brain/aws296 PMC3525058

[5] Iqbal N, Caswell H, Muir R, et al. Neuropsychological profiles of patients with juvenile myoclonic epilepsy and their siblings: an extended study. Epilepsia 2015;56:1301–1308. 26075864 10.1111/epi.13061

[6] Wandschneider B, Kopp UA, Kliegel M, et al. Prospective memory in patients with juvenile myoclonic epilepsy and their healthy siblings. Neurology 2010;75:2161–2167. 21048200 10.1212/WNL.0b013e318202010a

[7] Bailey JN, Patterson C, de Nijs L, et al. EFHC1 variants in juvenile myoclonic epilepsy: reanalysis according to NHGRI and ACMG guidelines for assigning disease causality. Genet Med 2017;19:144–156. 27467453 10.1038/gim.2016.86

[8] de Nijs L, Léon C, Nguyen L, et al. EFHC1 interacts with microtubules to regulate cell division and cortical development. Nat Neurosci 2009;12:1266–1274. 19734894 10.1038/nn.2390

[9] Meencke HJ, Janz D. The significance of microdysgenesia in primary generalized epilepsy: an answer to the considerations of Lyon and Gastaut. Epilepsia 1985;26:368–371. 4006898 10.1111/j.1528-1157.1985.tb05665.x

[10] Gottesman II, Gould TD. The endophenotype concept in psychiatry: etymology and strategic intentions. Am J Psychiatry 2003;160:636–645. 12668349 10.1176/appi.ajp.160.4.636

[11] Moran ME, Hulshoff Pol H, Gogtay N. A family affair: brain abnormalities in siblings of patients with schizophrenia. Brain 2013;136:3215–3226. 23698280 10.1093/brain/awt116 PMC3808683

[12] Potkin SG, Guffanti G, Lakatos A, et al. Hippocampal atrophy as a quantitative trait in a genome-wide association study identifying novel susceptibility genes for Alzheimer's disease. PLoS One 2009;4:e6501. 19668339 10.1371/journal.pone.0006501 PMC2719581

[13] Alhusaini S, Whelan CD, Sisodiya SM, Thompson PM. Quantitative magnetic resonance imaging traits as endophenotypes for genetic mapping in epilepsy. Neuroimage Clin 2016;12:526–534. 27672556 10.1016/j.nicl.2016.09.005 PMC5030372

[14] Alhusaini S, Kowalczyk MA, Yasuda CL, et al. Normal cerebral cortical thickness in first-degree relatives of temporal lobe epilepsy patients. Neurology 2019;92:e351–e358. 30587513 10.1212/WNL.0000000000006834

[15] Panizzon MS, Fennema-Notestine C, Eyler LT, et al. Distinct genetic influences on cortical surface area and cortical thickness. Cereb Cortex 2009;19:2728–2735. 19299253 10.1093/cercor/bhp026 PMC2758684

[16] Ecker C, Ronan L, Feng Y, et al. Intrinsic gray-matter connectivity of the brain in adults with autism spectrum disorder. Proc Natl Acad Sci USA 2013;110:13222–13227. 23878213 10.1073/pnas.1221880110 PMC3740833

[17] Sowell ER, Thompson PM, Leonard CM, Welcome SE, Kan E, Toga AW. Longitudinal mapping of cortical thickness and brain growth in normal children. J Neurosci 2004;24:8223–8231. 15385605 10.1523/JNEUROSCI.1798-04.2004 PMC6729679

[18] Dubois J, Benders M, Borradori-Tolsa C, et al. Primary cortical folding in the human newborn: an early marker of later functional development. Brain 2008;131:2028–2041. 18587151 10.1093/brain/awn137 PMC2724902

[19] Voets NL, Bernhardt BC, Kim H, Yoon U, Bernasconi N. Increased temporolimbic cortical folding complexity in temporal lobe epilepsy. Neurology 2011;76:138–144. 21148116 10.1212/WNL.0b013e318205d521 PMC3030232

[20] Ecker C, Ginestet C, Feng Y, et al. Brain surface anatomy in adults with autism: the relationship between surface area, cortical thickness, and autistic symptoms. JAMA Psychiatry 2013;70:59–70. 23404046 10.1001/jamapsychiatry.2013.265

[21] Winkler AM, Sabuncu MR, Yeo BTT, et al. Measuring and comparing brain cortical surface area and other areal quantities. NeuroImage 2012;61:1428–1443. 22446492 10.1016/j.neuroimage.2012.03.026 PMC3641659

[22] Hong SJ, Valk SL, Di Martino A, Milham MP, Bernhardt BC. Multidimensional neuroanatomical subtyping of autism spectrum disorder. Cereb Cortex 2018;28:3578–3588. 28968847 10.1093/cercor/bhx229 PMC7190887

[23] Worsley K, Taylor J, Carbonell F, et al. SurfStat: a Matlab toolbox for the statistical analysis of univariate and multivariate surface and volumetric data using linear mixed effects models and random field theory. Organ Hum Brain Mapp 2009;47(suppl 1):S102.

[24] Worsley KJ, Andermann M, Koulis T, MacDonald D, Evans AC. Detecting changes in nonisotropic images. Hum Brain Mapp 1999;8:98–101. 10524599 10.1002/(SICI)1097-0193(1999)8:2/3<98::AID-HBM5>3.0.CO;2-F PMC6873343

[25] Yeo BTT, Krienen FM, Sepulcre J, et al. The organization of the human cerebral cortex estimated by intrinsic functional connectivity. J Neurophysiol 2011;106:1125–1165. 21653723 10.1152/jn.00338.2011 PMC3174820

[26] Guerrini R, Dobyns WB. Malformations of cortical development: clinical features and genetic causes. Lancet Neurol 2014;13:710–726. 24932993 10.1016/S1474-4422(14)70040-7 PMC5548104

[27] Rakic P. Defects of neuronal migration and the pathogenesis of cortical malformations. Prog Brain Res 1988;73:15–37. 3047794 10.1016/s0079-6123(08)60494-x

[28] Hill J, Inder T, Neil J, Dierker D, Harwell J, Van Essen D. Similar patterns of cortical expansion during human development and evolution. Proc Natl Acad Sci USA 2010;107:13135–13140. 20624964 10.1073/pnas.1001229107 PMC2919958

[29] Shaw P, Kabani NJ, Lerch JP, et al. Neurodevelopmental trajectories of the human cerebral cortex. J Neurosci 2008;28:3586–3594. 18385317 10.1523/JNEUROSCI.5309-07.2008 PMC6671079

[30] Sur M, Rubenstein JLR. Patterning and plasticity of the cerebral cortex. Science 2005;310:805–810. 16272112 10.1126/science.1112070

[31] Vollmar C, O'Muircheartaigh J, Barker GJ, et al. Motor system hyperconnectivity in juvenile myoclonic epilepsy: a cognitive functional magnetic resonance imaging study. Brain 2011;134:1710–1719. 21616969 10.1093/brain/awr098 PMC3102244

[32] Bullmore E, Sporns O. The economy of brain network organization. Nat Rev Neurosci 2012;13:336–349. 22498897 10.1038/nrn3214

[33] Lewis DA, González-Burgos G. Neuroplasticity of neocortical circuits in schizophrenia. Neuropsychopharmacol 2008;33:141–165. 10.1038/sj.npp.1301563 17805309

[34] Wandschneider B, Centeno M, Vollmar C, et al. Motor co-activation in siblings of patients with juvenile myoclonic epilepsy: an imaging endophenotype? Brain 2014;137:2469–2479. 25001494 10.1093/brain/awu175 PMC4132647

[35] Giorgi FS, Guida M, Caciagli L, et al. Social cognition in juvenile myoclonic epilepsy. Epilepsy Res 2016;128:61–67. 27810518 10.1016/j.eplepsyres.2016.10.017

[36] Wandschneider B, Centeno M, Vollmar C, et al. Risk-taking behavior in juvenile myoclonic epilepsy. Epilepsia 2013;54:2158–2165. 24138327 10.1111/epi.12413 PMC4209120

[37] Lin JJ, Dabbs K, Riley JD, et al. Neurodevelopment in new-onset juvenile myoclonic epilepsy over the first 2 years. Ann Neurol 2014;76:660–668. 25087843 10.1002/ana.24240 PMC4362677

[38] Burgess PW, Gilbert SJ, Dumontheil I. Function and localization within rostral prefrontal cortex (area 10). Philos Trans R Soc Lond B Biol Sci 2007;362:887–899. 17403644 10.1098/rstb.2007.2095 PMC2430004

[39] Molenberghs P, Johnson H, Henry JD, Mattingley JB. Understanding the minds of others: a neuroimaging meta-analysis. Neurosci Biobehav Rev 2016;65:276–291. 27073047 10.1016/j.neubiorev.2016.03.020

[40] Li X, Lu ZL, D'Argembeau A, Ng M, Bechara A. The Iowa Gambling Task in fMRI images. Hum Brain Mapp 2010;31:410–423. 19777556 10.1002/hbm.20875 PMC2826566

[41] Camfield CS, Camfield PR. Juvenile myoclonic epilepsy 25 years after seizure onset: a population-based study. Neurology 2009;73:1041–1045. 19786695 10.1212/WNL.0b013e3181b9c86f

[42] Wandschneider B, Thompson PJ, Vollmar C, Koepp MJ. Frontal lobe function and structure in juvenile myoclonic epilepsy: a comprehensive review of neuropsychological and imaging data. Epilepsia 2012;53:2091–2098. 23106095 10.1111/epi.12003

[43] Atakli D, Soysal A, Atay T, Altintas H, Arpaci B, Baybas S. Somatosensory evoked potentials and EEG findings in siblings of juvenile myoclonic epilepsy patients. Epileptic Disord 1999;1:173–177. 10937150

[44] Suzuki T, Miyamoto H, Nakahari T, et al. Efhc1 deficiency causes spontaneous myoclonus and increased seizure susceptibility. Hum Mol Genet 2009;18:1099–1109. 19147686 10.1093/hmg/ddp006 PMC4817086

[45] Suzuki T, Delgado-Escueta AV, Aguan K, et al. Mutations in EFHC1 cause juvenile myoclonic epilepsy. Nat Genet 2004;36:842–849. 15258581 10.1038/ng1393

[46] Alhusaini S, Whelan CD, Doherty CP, Delanty N, Fitzsimons M, Cavalleri GL. Temporal cortex morphology in mesial temporal lobe epilepsy patients and their asymptomatic siblings. Cereb Cortex 2016;26:1234–1241. 25576532 10.1093/cercor/bhu315

[47] Knickmeyer RC, Gouttard S, Kang C, et al. A structural MRI study of human brain development from birth to 2 years. J Neurosci 2008;28:12176–12182. 19020011 10.1523/JNEUROSCI.3479-08.2008 PMC2884385

[48] Caciagli L, Bernasconi A, Wiebe S, Koepp MJ, Bernasconi N, Bernhardt BC. A meta-analysis on progressive atrophy in intractable temporal lobe epilepsy: time is brain? Neurology 2017;89:506–516. 28687722 10.1212/WNL.0000000000004176 PMC5539734

[49] Park KM, Kim TH, Han YH, et al. Brain morphology in juvenile myoclonic epilepsy and absence seizures. Acta Neurol Scand 2016;133:111–118. 25950250 10.1111/ane.12436

[50] Young NA, Szabó CÁ, Phelix CF, et al. Epileptic baboons have lower numbers of neurons in specific areas of cortex. Proc Natl Acad Sci USA 2013;110:19107–19112. 24191031 10.1073/pnas.1318894110 PMC3839763

